# Non-intubated video-assisted thoracoscopic surgery under combination of erector spinae plane block and thoracic paravertebral block

**DOI:** 10.1186/s12871-022-01634-4

**Published:** 2022-04-06

**Authors:** Ali Alagoz, Gokturk Findik, Hilal Sazak, Sevki Mustafa Demiroz, Ramazan Baldemir, Gulay Ulger, Musa Zengin

**Affiliations:** 1 Department of Anesthesiology and Reanimation, University of Health Sciences, Ankara Atatürk Chest Diseases and Thoracic Surgery Training and Research Hospital, Ankara, Turkey; 2 Department of Thoracic Surgery, University of Health Sciences, Ankara Atatürk Chest Diseases and Thoracic Surgery Training and Research Hospital, Ankara, Turkey; 3 Department of Thoracic Surgery, Gazi University Medical School, Ankara, Turkey

**Keywords:** Combination, Erector spinae plane block, Non-intubated video-assisted thoracoscopic surgery, NIVATS, Thoracic paravertebral block

## Abstract

**Background:**

The use of anesthetics and analgesic drugs and techniques in combination yields a multimodal effect with increased efficiency. In this case series, we aimed to evaluate the anesthetic effect of the thoracic paravertebral block (TPVB) and erector spinae plane block (ESPB) combination in patients, who underwent non-intubated video-assisted thoracoscopic surgery (NIVATS).

**Methods:**

Medical records of 16 patients, who underwent NIVATS for wedge resection under the combination of ESPB and TPVB were reviewed retrospectively. Demographic data of patients, duration of the sensory block, amount of the anesthetic agent used for premedication and sedo-analgesia, any presence of perioperative cough, operative times, postoperative visual analog scale (VAS) scores in the postoperative follow-up period, the need for additional analgesia, and patient satisfaction were reviewed.

**Results:**

Of the patients included in the study, 12 were men and 4 were women. The mean age was 48.6 years and the mean BMI was 24.7 kg/m^2^. The mean time needed for the achievement of the sensorial block was 14 min and the mean skin-to-skin operative time was 21.4 min. During the procedure, patients received 81.5 ± 27.7 mg of propofol and 30 ± 13.6 micrograms of remifentanil infusions, respectively. The mean dose of ketamine administered in total was 58.1 ± 12.2 mg. Only 2 patients needed an extra dose of remifentanil because of recurrent cough. No patients developed postoperative nausea vomiting. During the first 24 h, the VAS static scores of the patients were 3 and below, while VAS dynamic scores were 4 and below. Morphine consumption in the first postoperative 24 h was 13.2 mg.

**Conclusions:**

In conclusion, combined ESPB and TPVB with added intravenous sedo-analgesia in the presence of good cooperation between the surgical team and the anesthesiologist in the perioperative period can provide optimal surgical conditions including the prevention of cough in NIVATS. It is not sufficient to state that this combination is superior to alone ESPB or alone TPVB, as it is a preliminary study with a limited number of cases.

## Background

Video-assisted thoracoscopic surgery (VATS) has become a standard method for thoracic surgery and is associated with fewer complications compared to conventional thoracotomy [1]. Compared to the conventional method, VATS is a less invasive and traumatic surgical procedure, which improves patients’ outcomes [2]. The use of a double-lumen tube under general anesthesia is a standard procedure in patients undergoing VATS. However, intubated VATS (IVATS) is associated with several complications, such as hypoxemia because of single-lung ventilation, intubation-induced airway injury, ventilation-related lung injury, and postoperative nausea/vomiting [1, 3, 4].

It is an important advantage that non-intubated video-assisted thoracoscopic surgery (NIVATS) under spontaneous breathing with sedation and peripheral nerve block such as thoracic paravertebral block (TPVB), erector spinae plane block (ESPB), or serratus anterior plane block (SAPB) reduces potential risks of general anesthesia [3–7].

The first NIVATS procedures were reported by Vischnevski and Buckingham, who used local anesthesia and thoracic epidural anesthesia (TEA), respectively [8, 9]. Various local and regional analgesia techniques such as intercostal nerve blocks, ESPB, SAPB, TPVB, and TEA have been performed for NIVATS [5]. Previous studies have demonstrated that non-intubated thoracic surgery is a safe and feasible technique that allows rapid recovery [1, 10–12].

It is known that the use of anesthetics and analgesic drugs and techniques in combination yields a multimodal effect with increased efficiency [13]. In this case series, we aimed to evaluate the anesthetic effect of the TPVB and ESPB combination in patients, who underwent NIVATS.

## Methods

After receiving approval from Ankara Keçiören Training and Research Hospital, Clinical Research Ethics Committee (ID:2012-KAEK-15/2384, Date:12.10.2021), medical records of 16 patients, who underwent NIVATS under the combination of ESPB and TPVB in our hospital in the period between January 2021 and June 2021, were reviewed retrospectively. Patients who were considered for NIVATS had been consulted by thoracic surgeons to our clinic. The patients had been evaluated in the anesthesia clinic preoperatively. NIVATS had been planned for patients who were not expected to have a difficult airway in possible emergency intubation situations. Informed consent had been obtained from all patients in terms of anesthesia procedures in line with the routine practice of our clinic. Medical records of patients in the age range of 18-80 years and body mass index (BMI) range of 18-30 kg/m^2^, of patients assigned to American Society of Anesthesiologists (ASA) scores of I, II, III, and of patients, who underwent wedge resection with NIVATS were included in the study. Demographic data of patients, duration of the sensory block, amount of the anesthetic agent used for premedication and sedo-analgesia, any presence of perioperative cough, operative times, postoperative visual analog scale (VAS) scores in the postoperative follow-up period, the need for additional analgesia, and patient satisfaction information were reviewed and retrieved.

### Methods of performing nerve blocks

The anesthesia-analgesia and block procedures had been performed according to the routine practice of our clinic as stated in the following paragraphs.

After giving 0.03 mg/kg midazolam intravenously to patients for premedication, patients were transferred to the operating room. Standard monitorization was performed according to assigned ASA guidelines to patients. Patients were placed in the lateral decubitis position. Oxygen was administered through a nasal cannula at a rate of 2-4 l/min. Preemptive intravenous ketamine at a dose of 0.5 mg/kg was given for the alleviation of pain before performing the nerve block.

**Fig. 1:**
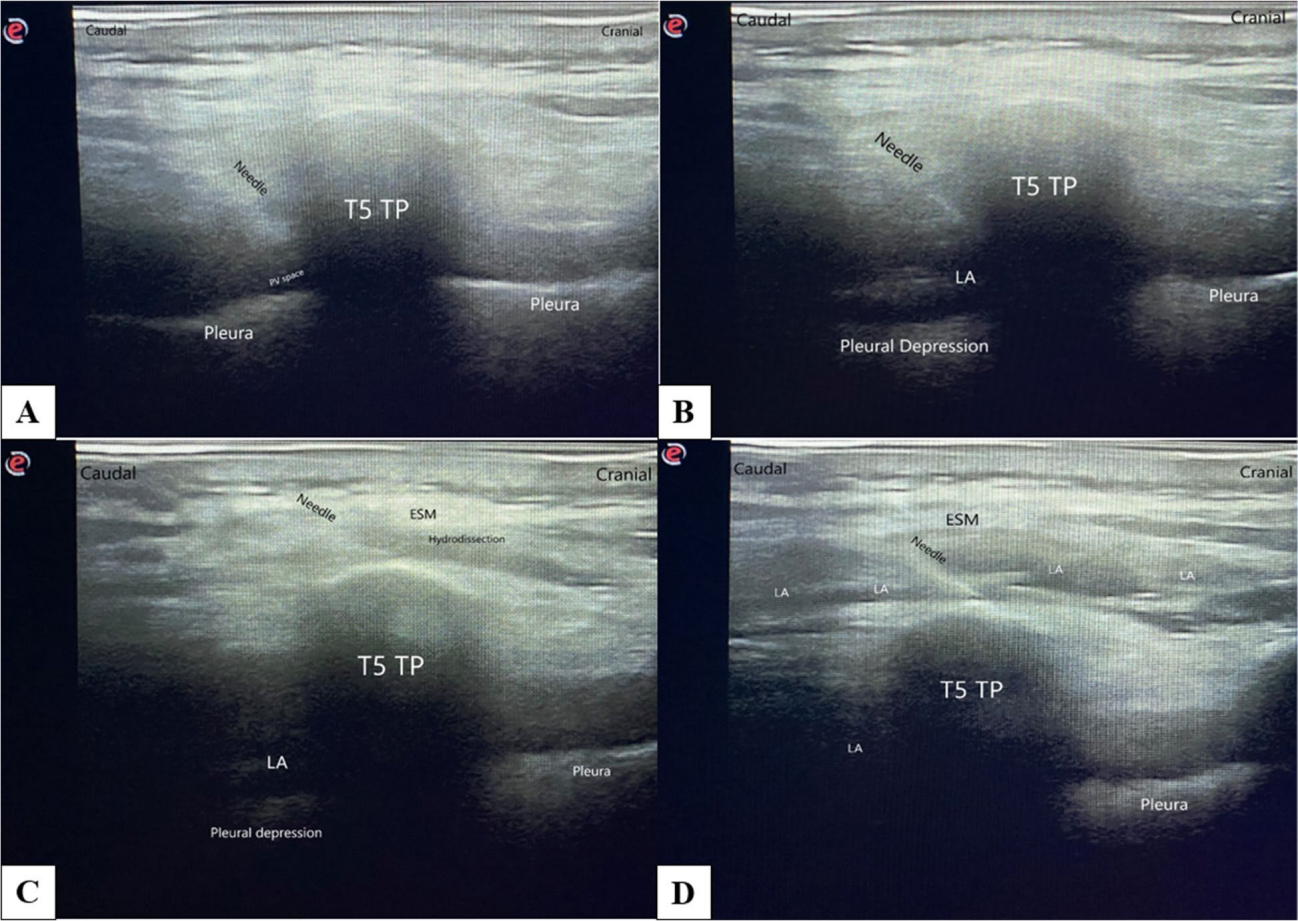
Anatomical view during Combination of Erector Spina Plane Block and Thoracic Paravertebral Block. **A** The view of the block needle in the paravertebral area before the block. **B** Ten ml of 0.25% bupivacaine and five ml of 2% lidocaine were administered and pleural depression was observed. **C** Hydrodissection with 2 ml of saline solution was performed into the interfascial plane above the transverse process and below the erector spinae muscle. **D** Ten ml of 0.25% bupivacaine and five ml of 2% lidocaine were administered beneath the erector spinae muscle. The local anesthetic spread caudally and cranially beneath the erector spinae muscle. (ESM: Erector spinae muscles; LA: local anesthetic; PV space: Paravertebral space; T: Thoracic; TP: Transverse process)

After providing skin antisepsis with povidone-iodine to the area, where the nerve block would be performed, the area was covered with sterile drapes. A 6-18 MHz linear probe (Esaote MyLab six, Genoa, Italy) covered by a sterile sheath was placed 2-3 cm lateral to the spinous process of the T (thoracic) 5 vertebra. The transverse process, muscles extending to the transverse process (the trapezius muscle, the rhomboid major muscle, and the erector spinae muscle), and the paravertebral space were visualized (Fig. 1a). Local anesthesia was performed by injecting 60 mg 2% lidocaine to the skin over the area, where the block would be performed. An ultrasound-compatible nerve block needle of 22-gauge and 8-mm (Pajunk, SonoPlexSTIM, Germany) was introduced 2-3 cm lateral to the spinous process of the T6 vertebra and advanced in the caudal-cranial direction using the in-plane technique. The needle was advanced to reach the paravertebral space at the level of the T5 vertebra. Then, 10 ml of 0.5% bupivacaine and 5 ml of 2% lidocaine were administered and the pleural depression was observed (Fig. 1b). Following this step, the needle was withdrawn from the paravertebral space and advanced at the T5 level over the transverse process and beneath the erector spinae muscle to reach the interfascial space. After hydrodissection (Fig. 1c) with 2 ml of normal saline and observing that the needle was placed in the correct location, 10 ml of 0.5% bupivacaine + 5 ml of 2% lidocaine was injected. The advancement of the local anesthetics caudally and cranially beneath the erector spinae muscle was observed (Fig. 1d). After performing the nerve block, sensorial block time was followed up by the pinprick test. Following the achievement of the sensorial block, surgery started. Sedoanalgesia was performed by administering propofol, ketamine, and remifentanil to patients during the surgery. The NIVATS procedure was started when a Ramsay sedation score of 3 was achieved.

At the end of the surgery, patients were transferred to the postoperative intensive care unit after 10 mg of metoclopramide was given intravenously. Intravenous morphine for 24 h was given to patients via the patient-controlled analgesia (PCA) method during their stay in the postoperative surgical intensive care unit. PCA was limited to 1 mg of bolus, 15 min of lockout, and a dose of 12 mg for 4 h. For multimodal analgesia, patients were given paracetamol 1 g intravenously every 8 h. Patients, with VAS scores of 4 or more, received tramadol 50 mg intravenously for additional analgesia. Postoperative static and dynamic VAS scores of patients were evaluated and noted in the postoperative 1st, 2nd, 6th, 12th, and 24th hours. Adverse events including the requirement for extra analgesia, allergic reactions, respiratory depression, sedation, urinary retention, nausea-vomiting, and pruritus were registered.

## Results

Data were analyzed from 16 patients, who underwent NIVATS under the combination of ESPB and TPVB in the period between January 2021 and June 2021. Of the patients included in the study, 12 were men and 4 were women. The mean age was 48.6 years and the mean BMI was 24.7 kg/m^2^. The mean time needed for the achievement of the sensorial block was 14 min and the mean skin-to-skin operative time was 21.4 min (Table 1).

**Table 1:** Demographic data, operative times, and sensory block time

Case No	Age (Year)	Gender	ASA	BMI kg/m^2^	Surgery time (minutes)	Sensory Block Time (minutes)	Surgical Side
1	63	M	2	26.4	30	14	L
2	22	M	2	21.7	18	13	L
3	65	M	3	24.8	24	16	R
4	67	M	3	26.6	20	15	L
5	32	F	2	21.3	28	18	R
6	49	M	3	28.1	20	13	R
7	73	M	2	23.1	16	14	R
8	56	F	2	24.4	22	12	R
9	39	M	2	28.5	14	14	L
10	23	M	1	26.3	14	12	R
11	21	M	1	19.2	21	16	R
12	61	M	2	27.4	28	14	R
13	62	F	2	23.8	24	12	R
14	70	M	3	25.6	24	13	L
15	30	M	2	22.7	22	16	L
16	46	F	2	26.3	18	13	R
Mean ± SD	48.6 ± 18.4	M/F (12/4)		24.7 ± 2.63	21.4 ± 4.81	14.0 ± 1.73	L/R (6/10)

Data presented as mean ± standard deviation

*Abbreviations*: *ASA* American Society of Anesthesiologist, *BMI* Body mass index, *F* Female, *L* Left, *M* Male, *R* Right

During the procedure, patients received 81.5 ± 27.7 mg of propofol and 30 ± 13.6 micrograms of remifentanil infusions, respectively. The mean dose of ketamine administered in total was 58.1 ± 12.2 mg. Only 2 patients needed an extra dose of remifentanil because of recurrent cough. No patients developed postoperative nausea-vomiting (Table 2).

**Table 2:** Mean analgesic consumption and adverse effects

Case No	Propofol (mg)	Remifentanil (mcg)	Ketamine (mg)	Cough
1	100	30	50	No
2	70	30	70	No
3	100	20	80	No
4	100	20	40	No
5	150	30	80	No
6	50	20	50	No
7	50	60	60	**Yes**
8	100	20	60	No
9	50	20	50	No
10	50	40	50	No
11	100	20	70	No
12	80	20	60	No
13	60	60	40	**Yes**
14	100	40	50	No
15	80	30	60	No
16	65	20	60	No
Mean ± SD	81.5 ± 27.7	30 ± 13.6	58.1 ± 12.2	2/16

Data presented as mean ± standard deviation

During the first 24 h, the VAS static scores of the patients were 3 and below, while VAS dynamic scores were 4 and below. VAS scores were lower in the first 6 h compared to the rest of the 24-h postoperative period. Morphine consumption in the first postoperative 24 h was 13.2 mg (Table 3).

**Table 3:** VAS score is and morphine consumption in the first postoperative 24 h

Case No	VAS (Static / Dynamic)					Postoperative Morphine consumption (mg)
	1st h	2nd h	6th h	12th h	24th h	
1	2/3	2/3	3/4	3/4	2/4	25
2	0/1	0/1	2/3	2/3	1/3	10
3	0/1	0/1	1/3	2/3	1/3	15
4	1/3	1/3	1/2	2/3	2/3	14
5	3/4	3/4	2/4	2/3	2/3	12
6	1/3	1/2	2/3	2/3	1/3	11
7	2/3	2/4	1/2	1/2	1/2	14
8	1/2	1/2	2/4	2/4	2/3	16
9	1/2	1/3	1/3	3/4	2/3	8
10	2/3	2/3	2/3	3/4	2/3	10
11	3/4	2/3	2/3	2/3	2/3	20
12	0/1	0/1	0/1	2/3	2/3	12
13	0/1	0/1	0/1	1/2	2/4	5
14	1/2	1/2	2/3	2/3	2/3	14
15	2/3	2/4	2/3	3/4	2/3	16
16	1/2	0/1	1/2	2/3	1/2	10
Mean	1.2/2.3	1.1/2.3	1.5/2.7	2.1/3.1	1.6/3	13.2

*Abbreviations*: *h* Hour, *VAS* Visual Analog scale

## Discussion and conclusion

Our study on patients undergoing NIVATS under the ESPB - TPVB combination and sedoanalgesia has shown that, with this technique, it is possible to achieve effective intraoperative anesthesia and postoperative analgesia with low complication rates and low morphine consumption.

NIVATS has become widely used in recent years [14]. NIVATS is considered appropriate, especially for high-risk patients, suggesting that this technique can help avoid potential risks of general anesthesia [14]. However, it is becoming preferred in low-risk patients, too, because it is associated with lower costs and fewer complications compared to those of general anesthesia [15]. Furthermore, avoiding general anesthesia is associated with many advantages such as rapid recovery, early oral intake, and early mobilization [15]. The obviation of the need for double-lumen tube use for single-lung ventilation in VATS has contributed significantly to the decrease in airway complications and potential adverse physiological effects following single-lung ventilation [16]. In our study, no patients needed intraoperative intubation and no hypoxia cases occurred. NIVATS performed with a combined block can prevent complications that may occur due to single lung ventilation provided with a double-lumen tube under general anesthesia.

One of the drawbacks of general anesthesia is the development of side effects of anesthetics and muscle relaxants. Of such side effects, postoperative nausea and vomiting are significant ones, which can impair patient comfort severely and which can lead to dehydration, deterioration of the nutritional status, and problems at the surgical incision site, consequently resulting in the prolongation of the hospital stay [17]. No patients developed nausea and vomiting in the postoperative period in our study. We think that the limited use of intravenously administered anesthetics for sedo-analgesia and the low morphine consumption in the postoperative period may act as important factors in the prevention of nausea and vomiting that may develop following NIVATS. Furthermore, we think that the antiemetic effect of propofol, which is used for sedation, contributes to the prevention of nausea and vomiting. Uncontrollable mediastinal movement and cough despite sedation may occur as major problems in patients undergoing NIVATS. Such untoward developments affect surgical manipulations unfavorably and may create a risk of trauma to vascular structures. Sedating the patient successfully in combination with the administration of narcotics may be effective in preventing the cough reflex [15]. In addition, the vagal block is a method to prevent cough reflex. In our study, we think that sedation along with remifentanil administration in adjunct to the ESPB + TPVB combination limited the development of cough reflex.

A consensus on patient selection for NIVATS has not been achieved yet [14, 16]. NIVATS was selected for high-risk patients initially but it has been increasingly used in low-risk patients in recent years [15]. Preoperative evaluation should be comprehensive for patient selection and the surgeon and anesthesiologist should discuss possible risks when deciding to perform NIVATS. In particular, patients with a risk of intubation difficulties should be evaluated to decide whether general anesthesia or NIVATS would be performed. The decision should be made based on anesthesiologist and surgeon experiences and the risks specific to the patient. We prefer general anesthesia in our clinic in patients, who do not consent to undergo NIVATS or for whom we predict difficult intubation.

Various opinions have been suggested on the choice of the anesthesia method and the block method to be applied to perform the NIVATS procedure. TEA, TPVB, intercostal block, or local anesthetic infiltration only are frequently used with intravenously administered anesthetics as adjuncts to these techniques in NIVATS procedures [7, 14, 18]. Piccioni et al. [7] used TPVB as a regional technique in two cases in which NIVATS was applied. They found that the use of thoracic paravertebral block in both cases resulted in adequate unilateral anesthesia, stable hemodynamics, and high patient satisfaction. Although studies indicate that TPVB is a method used in NIVATS, we could not find any RCT with clear results regarding the success rate of this block. Furthermore, we could not find any study, in which an ESPB and TPVB combination was applied with sedation for NIVATS. The purpose of performing these two types of nerve blocks together is to combine different mechanisms of action so that either ESPB or TPVB can compensate the other in case of a possible block failure. Low VAS scores up to the first postoperative 6 h in our study suggest that this combination of blocks is effective. At the same time, sedation applied with intravenously administered anesthetics has prevented potential anxiety and pain associated with the block procedure and served well to the aim of achieving adequate patient comfort during the surgery. No adverse events occurred in this study except for recurrent cough in only two patients in the perioperative period. Cough in these patients was suppressed by giving extra doses of remifentanil. In addition, effective analgesia was achieved in the first 24 h in the postoperative period. 

De Cassai et al. [19] evaluated the pharmacokinetics of lidocaine after bilateral ESP block, and it was defined that lidocaine has a rapid and extensive absorption rate. However, they observed that the peak concentrations did not reach the accepted toxicity limit after this block. In our study, we used a total of 20 ml of 0.5% bupivacaine and 10 ml of 2% lidocaine for unilateral combined ESPB and TPVB. These ratios used were more limited than the volume and concentration used by De Cassai et al., and local anesthesia systemic toxicity was not observed in any case.

Enhanced recovery after surgery (ERAS) has gained increasing popularity in thoracic anesthesia in recent years. ERAS protocols allow fast recovery and early hospital discharge with few complications [20, 21]. ERAS protocols are associated with many advantages such as low anesthetic consumption, effective analgesia, early oral intake, and early mobilization [20–22]. In this study, we observed that low consumption of intravenously administered anesthetics with the ESPB and TPVB combination limited the development of potential complications associated with general anesthesia and provided effective pain management in the intraoperative and postoperative periods. In addition, minimal postoperative morphine consumption limited the development of side effects such as respiratory depression and nausea/vomiting that might develop following opioid use. We think that this method can contribute to thoracic ERAS protocols.

There are some limitations of this study. First of all, the study was conducted with a limited number of patients. Only patients, who underwent wedge resection, were included in our study. Since the study is retrospective and preliminary, there is no standard sedation protocol. Sedative agents were administered by the anesthesiologist following the patient according to the patient’s hemodynamic and sedation status. There is a need for further prospective studies that would include other surgical techniques performed via VATS.

In conclusion, combined ESPB and TPVB with added intravenous sedoanalgesia in the presence of good cooperation between the surgical team and the anesthesiologist in the perioperative period can provide optimal surgical conditions including the prevention of cough in NIVATS. Furthermore, this combination contributes significantly to ERAS procedures in thoracic surgery by enabling the achievement of effective intraoperative anesthesia and postoperative analgesia. The combined ESPB and TPVB application in NIVATS is a new practice. It is not sufficient to state that this combination is superior to alone ESPB or alone TPVB, as it is a preliminary study with a limited number of cases. Large series of randomized controlled studies on this subject may be clarify this topic.

## Data Availability

The datasets generated and analyzed during the current study are not publicly available but are available from the corresponding author on reasonable request.

## Abbreviations

ASA: American Society of Anesthesiologists
BMI: Body mass index
ERAS: Enhanced recovery after surgery
ESPB: Erector spinae plane block
IVATS: Intubated video-assisted thoracoscopic surgery
NIVATS: Non-intubated video-assisted thoracoscopic surgery
PCA: Patient-controlled analgesia
SAPB: Serratus anterior plane block
T: Thoracic
TEA: Thoracic epidural anesthesia
TPVB: Thoracic paravertebral block
VAS: Visual analog scale
VATS: Video-assisted thoracoscopic surgery
